# Neural correlates of reward processing in healthy siblings of patients with schizophrenia

**DOI:** 10.3389/fnhum.2015.00504

**Published:** 2015-09-23

**Authors:** Esther Hanssen, Jorien van der Velde, Paula M. Gromann, Sukhi S. Shergill, Lieuwe de Haan, Richard Bruggeman, Lydia Krabbendam, André Aleman, Nienke van Atteveldt

**Affiliations:** ^1^Department of Educational Neuroscience and LEARN! Institute, VU University AmsterdamAmsterdam, Netherlands; ^2^CSI Lab, Department of Psychosis Studies, Institute of Psychiatry, King's College LondonLondon, UK; ^3^Neuroimaging Center, University Medical Center Groningen, University of GroningenGroningen, Netherlands; ^4^Department of Early Psychosis, Academic Psychiatric Centre, Amsterdam Medical CenterAmsterdam, Netherlands; ^5^Rob Giel Research Center, University Center for Psychiatry, University Medical Center Groningen, University of GroningenGroningen, Netherlands

**Keywords:** schizophrenia, siblings, reward processing, fMRI, default mode network (DMN)

## Abstract

Deficits in motivational behavior and psychotic symptoms often observed in schizophrenia (SZ) may be driven by dysfunctional reward processing (RP). RP can be divided in two different stages; reward anticipation and reward consumption. Aberrant processing during reward anticipation seems to be related to SZ. Studies in patients with SZ have found less activation in the ventral striatum (VS) during anticipation of reward, but these findings do not provide information on effect of the genetic load on reward processing. Therefore, this study investigated RP in healthy first-degree relatives of SZ patients. The sample consisted of 94 healthy siblings of SZ patients and 57 healthy controls. Participants completed a classic RP task, the Monetary Incentive Delay task, during functional magnetic resonance imaging (fMRI). As expected, there were no behavioral differences between groups. In contrast to our expectations, we found no differences in any of the anticipatory reward related brain areas (region of interest analyses). Whole-brain analyses did reveal group differences during both reward anticipation and reward consumption; during reward anticipation siblings showed less deactivation in the insula, posterior cingulate cortex (PCC) and medial frontal gyrus (MFG) than controls. During reward consumption siblings showed less deactivation in the PCC and the right MFG compared to controls and activation in contrast to deactivation in controls in the precuneus and the left MFG. Exclusively in siblings, MFG activity correlated positively with subclinical negative symptoms. These regions are typically associated with the default mode network (DMN), which normally shows decreases in activation during task-related cognitive processes. Thus, in contrast to prior literature in patients with SZ, the results do not point to altered brain activity in classical RP brain areas, such as the VS. However, the weaker deactivation found outside the reward-related network in siblings could indicate reduced task-related suppression (i.e., hyperactivation) of the DMN. The presence of DMN hyperactivation during reward anticipation and reward consumption might indicate that siblings of patients with SZ have a higher baseline level of DMN activation and possible abnormal network functioning.

## Introduction

Patients with schizophrenia often show motivational impairments and as a result fail to pursue goal-directed behavior, which particularly seems to be driven by aberrant DA functioning in reward-related brain areas (Kapur et al., 2005; Howes and Kapur, 2009; Hartmann et al., 2014). Despite apparently normal hedonic experiences, patients rarely engage in behavior that is directed to obtaining rewards and gaining pleasure (Strauss et al., 2014). This is suggestive of aberrant reward processing in schizophrenia. Rewards are desirable outcomes that serve to influence behavior and therefore reward processing is one of the most basic and important mechanisms for guiding human behavior and managing everyday life encounters successfully. Research in animals and in the healthy human population has established the crucial role for mesolimbic dopamine in reward processing (Schultz, 1997, 1998, 2006; Elliott et al., 2000; Bayer and Glimcher, 2005; Pizzagalli et al., 2008). Dopamine is essential in motivated learning, reinforcement learning, assigning salience, reward anticipation and the associated reward prediction error (Robbins and Everitt, 1996; Schultz, 1997; Bayer and Glimcher, 2005; Juckel et al., 2006b; Drew et al., 2007).

Dopaminergic projections in the mesolimbic system and the fronto-striatal network arise in the ventral tegmental region and project to the prefrontal cortex (PFC) via the dorsal and ventral striatum (VS) (Kapur, 2003; Drew et al., 2007). Projections from the ventral tegmental area/substantia nigra (VTA/SN) to the VS are central in the assignment of salience and reward (Zink et al., 2004; Berridge, 2007), which causes attentional and behavioral resources to be redirected. Aberrant cortical-striatal functions and interactions may be associated with dysfunctions in different stages of reward processing. Two stages can be distinguished: reward anticipation (i.e., expecting to receive a reward) and reward consumption (i.e., receiving the actual reward). Neuroimaging research in healthy individuals has implied that these stages are based on different brain regions: reward anticipation has been linked to activation in a number of limbic regions, in particular the VS (Knutson et al., 2001a; Bjork et al., 2010; Rademacher et al., 2010; Urban et al., 2012), the anterior cingulate cortex (ACC) and VTA/SN, whereas frontal regions, more specifically the medial prefrontal cortex (mPFC) and the right dorsolateral prefrontal cortex (dlPFC), have been associated with reward consumption (Knutson et al., 2001b, 2003).

A number of studies have been conducted to investigate reward processing in schizophrenia, but the findings up to now are inconclusive. Performance on reward processing tasks sometimes indicates that patients are slower, demonstrated by reduced reaction times in patients compared to controls (Schlagenhauf et al., 2008; Nielsen et al., 2012b). Several neuroimaging studies indicate that unmedicated patients with schizophrenia and patients on atypical antipsychotics show less striatal activity during reward anticipation in a widely used reward processing task, the Monetary Incentive Delay task, than healthy controls (Juckel et al., 2006a,b; Kirsch et al., 2007; Schlagenhauf et al., 2008, 2009; Nielsen et al., 2012a,b). In contrast, several studies have shown a normal neural reward anticipation response in patients with schizophrenia on atypical antipsychotics (Juckel et al., 2006a,b; Schlagenhauf et al., 2008; Walter et al., 2009; Simon et al., 2010; Nielsen et al., 2012a). Studies that investigated the reward consumption phase found no differences between healthy controls and patients with schizophrenia (Simon et al., 2010; Nielsen et al., 2012b). This suggests that in particular reward anticipation, i.e., “wanting,” seems to be impaired in schizophrenia rather than the consumption of reward, i.e., “liking” (Gard et al., 2007; Kirsch et al., 2007; Nielsen et al., 2012b). Moreover, neural correlates of reward processing seem to be associated with symptom severity in patients with schizophrenia. A negative correlation between VS activation and positive symptoms was found by Nielsen et al. (2012b) and negative symptoms were shown to be negatively correlated with VS activation (Robbins and Everitt, 1996; Juckel et al., 2006a,b; Schlagenhauf et al., 2008; Simon et al., 2010). More specifically, anhedonia is one of the symptoms that seems to be related to a decrease in activation in the striatum (Juckel et al., 2006a,b). Previous research indicates that negative symptoms may predict future onset of schizophrenia and illness outcome (Gooding et al., 2005).

Although research on patients is vital and yielded substantial knowledge so far, these results cannot clarify the contribution of the genetic risk to abnormal reward processing in schizophrenia. The strongest known predictor of a disorder in the schizophrenia spectrum is the presence of an affected first-degree relative. This genetic risk accounts for 8–12 times increased risk (Kendler and Diehl, 1993; Stone et al., 2005; MacDonald et al., 2009). Moreover, behavioral and especially neuroimaging findings from patients are difficult to interpret, due to D2 blocking effects of antipsychotic medication, which changes striatal activation (Pessiglione et al., 2006), chronic hospitalization, and the potential neurotoxic effects of schizophrenia (MacDonald et al., 2009). Only two recent studies have investigated reward processing in relatives of patients so far, reporting blunted VS activity in siblings (de Leeuw et al., 2015) and other first-degree relatives (Grimm et al., 2014), in the absence of behavioral differences. The present study includes a larger homogeneous first-degree relatives (siblings) sample using a neutral modified version of the MID task.

The aim of the present study was to examine the underlying neural mechanisms of reward processing in siblings of patients with schizophrenia. Insight into neural correlates of reward processing in healthy siblings of schizophrenia patients may shed light on the possible underlying vulnerability and provide more insight on reward processing throughout the spectrum of the disorder. We expected to find: (a) no behavioral differences between the groups in terms of accuracy and reaction times on the reward processing task; (b) reduced activation of the VS, ACC, and VTA/SN during reward anticipation in siblings compared to controls; (c) no differences in the mPFC and the right dlPFC during the consumption of reward in siblings vs. controls, (d) blunted fMRI responses in the anticipation phase are expected to be negatively correlated with subclinical symptom severity (in particular negative symptomatology). To investigate this, we acquired functional magnetic resonance imaging (fMRI) data on 94 healthy siblings of patients with schizophrenia and 57 healthy controls while participating in a monetary incentive delay (MID) task tapping into reward anticipation and reward consumption.

## Methods

### Subjects

The subjects were 94 healthy siblings of patients with schizophrenia, and 57 healthy control subjects. The sample (*N* = 151) was recruited from a multi-center (Amsterdam, *N* = 81; Groningen, *N* = 70) add-on study from the Dutch Genetic Risk and Outcome in Psychosis (GROUP) project (https://www.group-project.nl) and had an age range between 18–60 years. Inclusion criteria for all subjects were: able and willing to give an informed consent and a good command of the Dutch language. The main exclusion criterion for the control group, and not for the sibling group, was: a personal or family history of a psychotic disorder. For a more detailed overview of participant inclusion for the GROUP study see Korver et al. (2012). Further exclusion criteria consisted of MRI contraindications such as metal implants, prostheses, pregnancy, and history of claustrophobia or epilepsy. The study was approved by the local ethics committees in Amsterdam and Groningen and conducted with strict compliance to ethical standards.

### Experimental design

**Figure 1:**
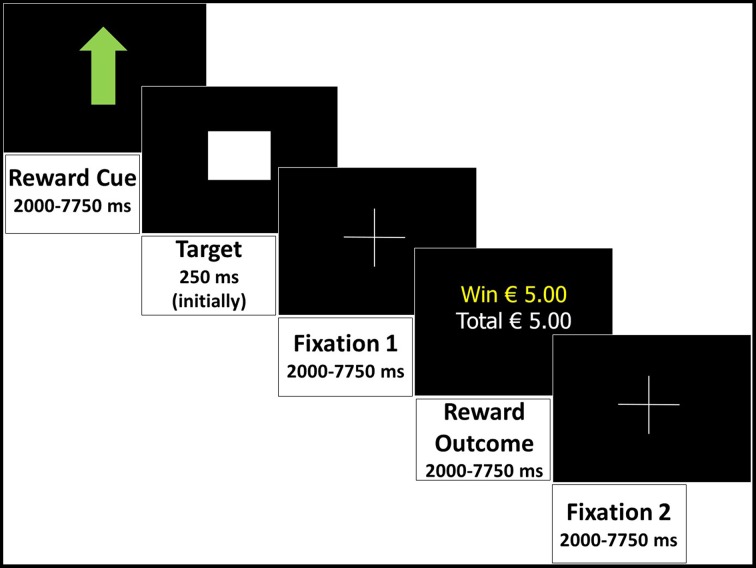
**Monetary Incentive Delay task: an example of a successful large reward trial displaying all events and durations in the trial**.

Subjects completed a modified version of the Monetary Incentive Delay (MID) task (Knutson et al., 2001b). In the current version, three levels of reward anticipation were visually cued: large reward, small reward, or no reward (control trial). The participants' reaction time of a button press to a target determined whether the reward outcome was successful (within the target presentation time) in each trial. The two phases of interest are the anticipatory phase (during the cue presentation); and the outcome phase (trial outcome presentation). Event durations were randomly assigned to the trials in each run, but equal for each participant, and summing up to a fixed total trial duration of 18.5 s (semi-random jittered). Each trial (Figure 1) consisted of (1) reward cue (semi-random jittered duration: 2000–7750 ms): a large green arrow pointing up indicating a potential reward of € 5 (large reward), a small green arrow pointing up indicating potential reward of € 0.5 (small reward), or a small green arrow pointing up combined with a red one pointing down (control trial, no reward), (2) target: presentation of a brief “target” square (duration initially 250 ms, then adjusted by an algorithm to ensure 66% success), (3) fixation 1: a delay of 2000–7750 ms (semi-random jittered), (4) reward outcome: presentation of the outcome of the trial (semi-random jittered 2000–7750 ms), (5) fixation 2 (semi-random jittered 2000–7750 ms).

All participants performed three runs of 31, 32, and 33 trials each, in randomized order. Half of the 96 trials were control trials (48) and the other trials were equally divided between large and small reward (24 each). One run lasted approximately 8 min, depending on how many trials there were in the run. Instructions were given to the participants about the different reward symbols and they were requested to respond as fast as possible to the target in all trials by pressing a button. To increase their motivation, participants were told that if they performed above average, they could earn a bonus of € 5. For ethical reasons, all participants received this bonus at the end of the session. In addition to this bonus, participants were given a voucher of € 25 for their participation regardless of their performance.

### Subclinical symptom assessment

The Community Assessment of Psychic Experience (CAPE) is a questionnaire based on self-report (http://www.cape42.homestead.com). It measures the lifetime prevalence of psychotic-like experiences in the general population. Results from previous research indicates that these self-reported psychotic-like experiences are stable, valid, and reliable (Konings et al., 2006). The questionnaire consists of 42 items within three dimensions: positive (20 items), negative (14 items) and depressive symptoms (8 items). We focused on the positive and negative scale in our study. The CAPE answers are rated on two 4-point Likert scales; one to indicate the frequency of symptoms and one to indicate the distress the symptoms inflict; in our study we focused on the frequency scores and not the subjective distress scores. The CAPE scores provide a total score per dimension by adding the score of each item in these dimensions (item scores range from 1 to 4), divided by the amount of items filled in. In this study, the Dutch translation of the CAPE was used.

### Scanning parameters

Imaging data were acquired on two 3.0 Tesla whole body scanners (Philips Intera, Best, NL) located at the University Medical Center Groningen and at the Academic Medical Centre in Amsterdam. Both systems were equipped with an 8-channel SENSE head coil and the scanning parameters were set in an identical way. Foam padding was placed around the subject's head in the coil to minimize head movement. The functional images were acquired by a T2^*^-weighted echo-planar imaging sequence scanning 40 axial slices of 2.4 mm thick with 1 mm gap, providing complete brain coverage. The in-plane resolution was 2.8 × 2.8 mm (FOV 224 × 224), TR = 2.00 s; TE = 25 ms. There were 258 volumes per run. For anatomical reference, a T1-weighted image (170 slices; isotropic voxels of 1 mm; TR 9 ms; TE 3.54 ms; α 8°; FOV 256 mm) was acquired in the bicommissural plane, covering the whole brain. Physiological data (ECG) during the task were also measured, but these data are not included in the current analyses as they are beyond the scope of this report.

### Statistical analyses

The demographical and behavioral data were analyzed using SPSS, version 20 (SPSS, IBM, Chicago, Illinois). Differences in group characteristics were examined with independent samples *t*-tests. Since the subclinical symptom scores have a non-normal distribution they were examined with Mann-Whitney *U*-tests. Accuracy (i.e., percentage of successful, on time button presses) and reaction time (RT, i.e., time between target presentation and button press, for all responses) were used as dependent variables, and analyzed by the ANCOVA module, with group (siblings, controls) as fixed factor and scanning site as covariate. Separate analyses were run for large and small reward trials. In addition, we used paired-sample *t*-tests to see whether the task showed an effect of reward on reaction times and accuracy. Linear regression analyses with group, age and scan site as predictors were performed to examine the effect of age on the behavioral measures.

The imaging data was analyzed using Brainvoyager QX, version 2.8 (Brain Innovation, Maastricht, The Netherlands). Preprocessing of the functional time-series consisted of slice scan-time correction, 3D motion correction, temporal highpass filtering (0.01 Hz) including linear trend removal, and modest temporal Gaussian smoothing (FWHM = 3 s). Finally, spatial smoothing using a 3D Gaussian kernel (FWHM = 6 mm) was performed. The preprocessed functional data were then coregistered to each individual anatomical scan, and resampled to 3 × 3 × 3 mm resolution in Talairach space resulting in normalized 4D volume time-course data. For each run of each subject, a stimulation protocol was created defining the onsets and offsets of the events. Using these protocols, design matrices were computed by convolving each event with a standard hemodynamic response function. The final design matrices contained 6 predictors of interest: reward cue (3x: control, small, large), indicating the anticipation of reward, and reward outcome + fixation 2 (3x: win, no win, control), indicating the consumption of reward. Additionally, there were 6 predictors-of-no-interest: target (3x: control, small, large) and fixation 1 (3x: control, small, large).

#### Whole-brain analyses

To increase power and to look at overall patterns of reward processing, large and small reward trials were combined for the whole-brain analyses. A voxel-wise, random-effects, whole-brain General Linear Model (GLM) was run to (1) identify the general activation pattern evoked by reward processing in the used task, by contrasting the reward anticipation (small + large reward combined) and reward consumption (small + large reward combined, where subjects actually won) events with the control trials (Bonferroni corrected at *p* < 0.001) and (2) to explore group differences in reward processing. To further examine group effects, a second-level analysis of covariance (ANCOVA) was run on the reward anticipation and consumption predictors (small + large combined; for analyses investigating trial differences see Supplementary Material), with group as between factor, and scanning site as covariate. To correct for multiple comparisons, a cluster-extent threshold (alpha < 0.05) determined by Monte Carlo simulations with 1000 iterations was applied to the resulting maps (Forman et al., 1995) based on an initial threshold of *p* < 0.01 (Goebel et al., 2006) across the whole-brain volume. For the group*anticipation map, the resulting spatial cluster threshold was 31 voxels/812 mm^3^. For the group*outcome map, the resulting spatial cluster threshold was 18 voxels/460 mm^3^. For each active cluster remaining after applying this threshold, individual beta weights (separately for small reward and large reward during the anticipation phase, and separately for win trials during the outcome phase) were extracted for visualization of the direction of the group differences, the differences between large and small reward and to investigate correlations with the symptom scores.

#### ROI-based analyses

We complemented the whole-brain analyses with analyses using a priori defined regions-of-interest (ROI's; Table 1 in Supplementary Material), based on the Talairach coordinates from previous research (Nielsen et al., 2012b). Different regions were used as ROI's for the reward anticipation and reward consumption phases, due to the finding that these two phases recruited distinct brain regions in previous research (Knutson et al., 2003). For the reward anticipation phase, four regions—the VTA/SN, right and left ventral striatum (VS), and anterior cingulate cortex (ACC)—were defined in line with their known role during reward anticipation. For the reward consumption phase, two regions—the medial prefrontal cortex (mPFC) and right dorsolateral prefrontal cortex (DLPFC)—were defined, due to their association with evaluation of rewarding outcomes. The ROI's for the smaller brain regions (i.e., left and right VS, and VTA/SN) were created with a 5 mm sphere centered around the published coordinates. For the larger brain regions (i.e., ACC, mPFC, and DLPFC) 10 mm spheres were used. ANCOVA analyses with group as between-subjects factor and scanning site as a covariate were run, on the averaged time-courses per ROI, using the individual design matrices. The results of the ROI-based analyses were corrected at alpha levels of 0.0125 per test (0.05/4; Bonferroni corrected) for regions tested during reward anticipation (4 ROI's) and 0.025 per test (0.05/2; Bonferroni corrected) for regions tested during reward consumption (2 ROI's). For each ROI, individual beta weights were extracted to investigate correlations with symptom scores.

## Results

### Demographics

The demographic group characteristics are displayed in Table 1. There were no significant differences between siblings and controls in terms of gender [*t*_(149)_ = 1.49, *p* = 0.137], education [*t*_(149)_ = −1.02, *p* = 0.312], and handedness [*t*_(149)_ = 0.23, *p* = 0.816]. There was a significant group difference for age [*t*_(149)_ = 2.77, *p* = 0.006], with controls having a higher mean age than siblings (respectively being 36 years and 32 years). There were no significant group differences between CAPE scores; positive dimension (*z* = −0.837, *p* = 0.402, with a mean rank of 65.52 for siblings and 71.28 for controls), negative dimension (*z* = −0.263, *p* = 0.792, with a mean rank of 66.86 for siblings and 68.72 for controls).

**Table 1:** **Participant characteristics**.

	**Siblings (*****N*** **= 94)**		**Control group (*****N*** **= 57)**	
	**Mean/N**	**SD/%**	**Mean/N**	**SD/%**
**AGE**				
	36.4	10.1 (range 22–55 years)	32.2	8.4 (range 20–59 years)
**GENDER**				
Men	41	43.6	32	56.1
Women	53	56.4	25	44.9
**HANDEDNESS**				
Right	77	81.9	48	84.2
Mixed	2	2.1	8	14
Left	15	16	1	1.8
**EDUCATION**				
University (academic)	26	27.7	21	36.8
University (vocational)	38	40.4	14	38.6
Vocational education	26	27.7	12	21
High school	4	4.3	2	3.6
**CAPE SCORES**				
Positive dimension (frequency)	1.10	0.13	1.13	0.18
Negative dimension (frequency)	1.44	0.39	1.45	0.37
**TASK ACCURACY (% HITS)**				
Control trial	43.62	15.25	42.32	14.31
Small reward trial	58.38	15.33	59.84	14.64
Large reward trial	57.34	14.44	57.42	15.04
**TASK REACTION TIME (ms)**				
Control trial	291.07	40.45	291.99	34.62
Small reward trial	272.77	30.96	269.29	24.75
Large reward trial	269.36	31.52	269.19	27.06

### Behavioral results

The percentage of hits (accuracy) and the RTs are displayed in Table 1. There were no significant differences between siblings and controls for either the large reward trials, in terms of accuracy [*F*_(1, 148)_ = 0.001, *p* = 0.975] or RT [*F*_(1, 148)_ = 0.02, *p* = 0.897], small reward trials [*F*_(1, 148)_ = 0.369, *p* = 0.544; *F*_(1, 148)_ = 0.77, *p* = 0.381] or control trials [*F*_(1, 146)_ = 0.154, *p* = 0.695; *F*_(1, 146)_ < 0.001, *p* = 0.993], respectively. A linear regression model with age, group and scan site as predictors showed no significant effect of age on any of the behavioral measures (see Supplementary Material for statistical tests). There was a significant difference between control trials and reward trials on reaction times in siblings and controls [control trials > small reward trials: respectively, *t*_(92)_ = 9.054, *p* < 0.001, *t*_(55)_ = 7.529, *p* < 0.001; and control trials > large reward trials: *t*_(92)_ = 9.311, *p* < 0.001, *t*_(55)_ = 7.177, *p* < 0.001].

### fMRI results

#### Whole-brain analyses: Overall task effect

**Figure 2:**
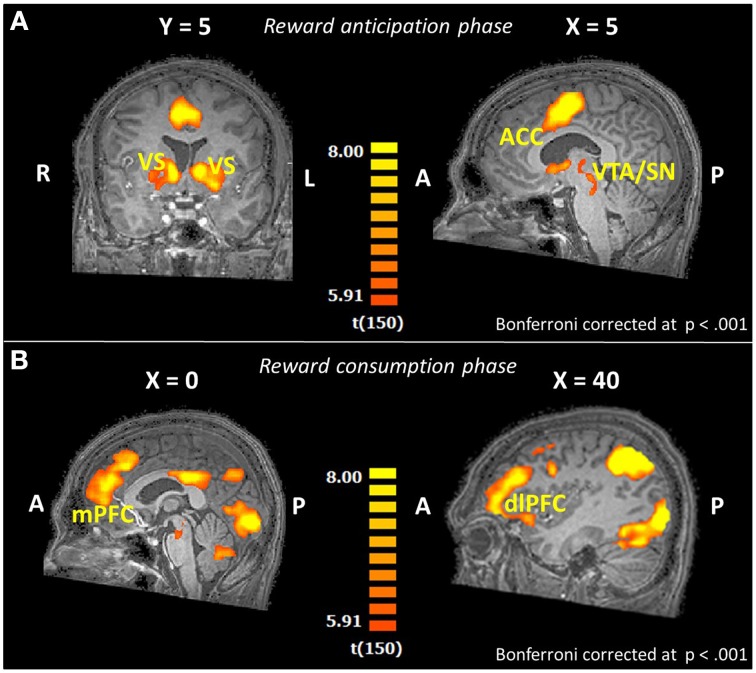
**Brain activation patterns for the whole-brain overall task effect (reward trials > control trials; whole-brain GLM; ***N*** = 151) shown on an anatomical scan of one of the control participants (A) in the reward anticipation phase for overall reward (small and large reward together), (B) in the reward consumption phase (win trials > controls trials; ***N*** = 151)**. VS, ventral striatum; ACC, anterior cingulate cortex; VTA, ventral tegmental area; SN, substantia nigra; mPFC, medial prefrontal cortex; dlPFC, dorsolateral prefrontal cortex.

First, we evaluated the overall task effect to see if the task produced the expected activation patterns during reward processing. In Figure 2A the regions active for the whole sample during the anticipation of reward (any kind of reward; small or large reward), contrasted with the control trials without reward, are displayed. We observed robust activation in important reward-related areas; a substantial region in and near the caudate, including the VS, a sizable region at the red nucleus containing part of the SN, and the mid/anterior cingulate cortex (Table 2 in the Supplementary Material). For the outcome phase, areas relevant in reward consumption showed activation, including a wide range of other frontal brain areas and, more importantly, including reward-related areas such as parts of the mPFC and the right dlPFC (Figure 2B, and Table 3 in the Supplementary Material).

#### Whole-brain analysis: Group effects

**Figure 3:**
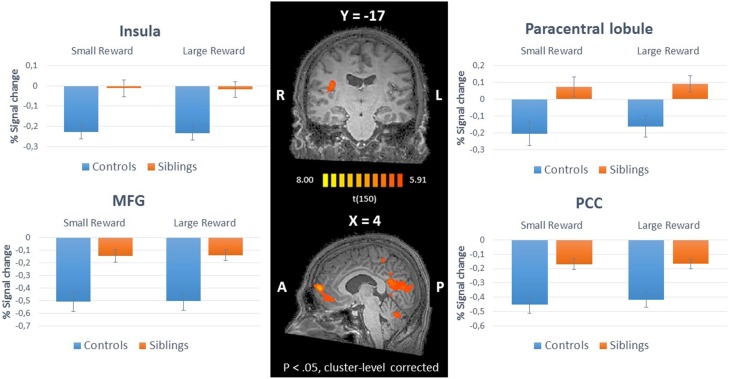
**Inner panel:** Group differences during anticipation of reward (ANCOVA; *N* = 151) depicted on an anatomical scan of one of the control participants (*p* < 0.05, cluster-level corrected). **Outer panel**: The difference in percent signal change between small and large rewards in these regions for siblings (*N* = 94) and controls (*N* = 57). MFG, medial frontal gyrus; PCC, posterior cingulate cortex.

**Figure 4:**
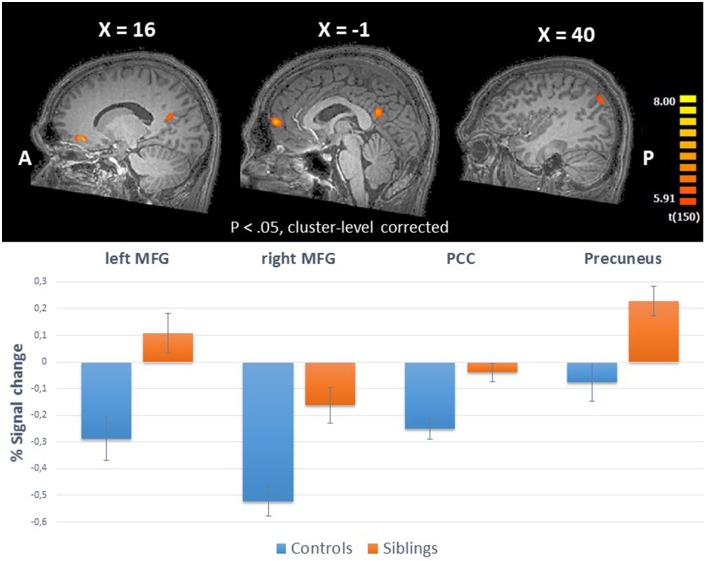
**Upper panel:** The whole-brain group effects during win trials in the reward consumption phase (ANCOVA; *N* = 151) displayed on an anatomical scan of one of the control participants (*p* < 0.05, cluster-level corrected). **Lower panel**: Group differences (% signal change) between siblings (*N* = 94) and controls (*N* = 57) during consumption of reward in the regions with a whole-brain group effect. MFG, medial frontal gyrus; PCC, posterior cingulate cortex.

The whole-brain ANCOVA showed an effect of group during the reward anticipation phase in several brain regions: the insula, posterior cingulate, medial frontal gyrus, and the paracentral lobule (Table 2, Figure 3). The extracted beta-weights indicate that the anticipation of reward was associated with less deactivation in siblings compared to controls in the insula, posterior cingulate and MFG, and a positive activation compared to deactivation in the paracentral lobule (bar graphs in Figure 3). Within these brain regions percent signal change for small and large reward are displayed separately, but they were not different in any of the regions (see Supplementary Material for statistical tests). The whole-brain ANCOVA showed an effect of group during the reward consumption phase within the bilateral medial frontal gyrus, posterior cingulate and the right precuneus (Table 3, Figure 4), again with siblings showing less deactivation than controls in the time period when subjects received their rewards.

**Table 2:** **Brain areas with a significant main effect of group during anticipation of reward**.

Cerebral Regions^*^	**Hemisphere**	**Brodmann area**	**Talairach coordinates**			**Cluster size**
**Siblings > Controls**			**x**	**y**	**z**	**Nr. Of voxels (mm^3^)**
***Reward Anticipation > Baseline***						
Insula	Right	13	38	−18	20	911
Posterior Cingulate	Right	23	2	−63	15	14,086
Medial Frontal Gyrus	Right	10	3	52	6	4869
Paracentral Lobule	Left	5	−1	−40	51	1204

* *Corrected cluster-level threshold of p < 0.01*.

**Table 3:** **Brain areas with a significant main effect of group during the consumption of reward**.

**Cerebral Regions**	**Hemisphere**	**Brodmann area**	**Talairach coordinates**			**Cluster size**
**Siblings > Controls**			**x**	**y**	**z**	**Nr. Of voxels (mm^3^)**
***Outcome Win > Baseline***						
Precuneus	Right	39	42	−69	33	514
Posterior Cingulate	Right	23	4	−55	19	2596
Medial Frontal Gyrus	Right	10	11	40	−4	1152
Medial Frontal Gyrus	Left	10	−1	57	12	1001

^*^Corrected threshold of p < 0.01.

#### ROI analyses

A priori defined ROI's were analyzed separately for the reward anticipation phase and the consumption phase. For the reward anticipation, there were no significant group differences for the left VS: for small reward *F*_(1, 148)_ = 2.09, *p* = 0.151, and large reward *F*_(1, 148)_ = 1.88, *p* = 0.172, right VS: for small reward *F*_(1, 148)_ = 0.08, *p* = 0.772, and large reward *F*_(1, 148)_ = 0.79, *p* = 0.377, ACC: small reward *F*_(1, 148)_ = 0.37, *p* = 0.542, and large reward *F*_(1, 148)_ = 0.80, *p* = 0.372, VTA/SN: small reward *F*_(1, 148)_ = 1.16, *p* = 0.283, and large reward *F*_(1, 148)_ = 0.82, *p* = 0.368. For the win trials in the outcome phase, no significant group difference were found in the mPFC *F*_(1, 148)_ = 1.95, *p* = 0.165, and the dlPFC *F*_(1, 148)_ = 0.21, *p* = 0.647.

### Correlations with symptoms

**Figure 5:**
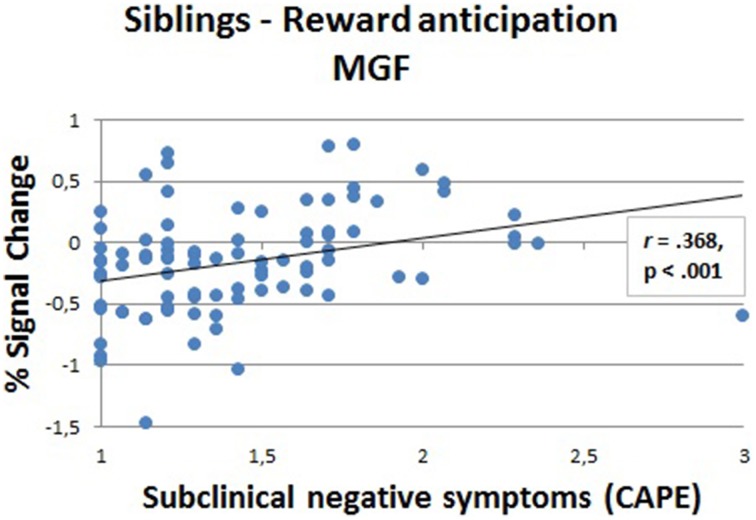
**Correlation (Spearman's rank) between fMRI response strength and subclinical negative symptoms in siblings (***N*** = 88) in the medial frontal gyrus (MFG) during the anticipation phase (large reward)**.

In the regions that showed an effect of group in the whole-brain analyses (see Figures 3, 4), we calculated the correlation between the fMRI response strength (beta estimates) and the CAPE scores (Figure 5). We eliminated extreme outliers (mean ± 2 sd) in beta values to ensure that possible correlations were not driven by outliers. Due to non-normal distribution of the subclinical symptom scales, we performed Spearman's rank correlations. To correct for multiple comparisons in the whole-brain areas, correlations were corrected at alpha levels of 0.00625 per test (0.05/8, Bonferroni corrected). In siblings, there was a positive correlation between MFG activation (large reward trials), where a group effect of reward anticipation was found, and negative symptoms (*r* = 0.368, *p* < 0.001).

Likewise, we calculated correlations between subclinical symptoms and fMRI response strength in the anticipation phase in the a priori defined ROIs (corrected at alpha levels of 0.0125, Bonferroni corrected; 0.05/4). For siblings and for controls, no correlations between brain activity and symptom scores were found in the a priori defined ROIs.

## Discussion

The aim of this study was to investigate the underlying neural mechanisms of reward processing in siblings of patients with schizophrenia. First, we confirmed the behavioral effect of reward in the MID task and the absence of behavioral differences between groups, as was in line with previous research (Grimm et al., 2014; de Leeuw et al., 2015). In addition, we confirmed that the task activated the intended reward-related brain regions during anticipation and consumption of reward.

In the reward anticipation phase, we did not find the expected blunted response in the VS in siblings or in the other a priori defined ROIs (VTA/SN and ACC). This is in contrast with earlier findings in siblings and other first-degree relatives (Grimm et al., 2014; de Leeuw et al., 2015), these different findings could be due to group differences, as we had a relatively healthy homogeneous sibling sample, and slight task differences, as our task was a neutral modified version of the MID task. The whole-brain analyses, however, revealed differences between groups during the anticipation of reward in the insula, the PCC, the MFG and the paracentral lobule. Looking closer at these activations, siblings apparently showed less deactivation compared to healthy controls in the insula, the PCC, the MFG. This lack of deactivation in the PCC and the medial frontal region may be related to abnormalities in the default mode network (DMN), which includes these areas (Raichle et al., 2001). Research in healthy subjects on the DMN indicates that the mPFC is activated during construction of mental simulations and receives input from the medial temporal lobe. Information from these two regions may subsequently be integrated by the PCC (Buckner et al., 2008). This network has been regarded as an underlying network in mental mind wandering/mental time travel, theory of mind and perspective taking; all constructions of complex self-referential stimuli (Buckner and Carroll, 2007; Mason et al., 2007; Molnar-Szakacs and Arzy, 2009). Normally, the DMN demonstrates decreases in activation during task-related cognitive processes (Buckner et al., 2008). In patients with schizophrenia, aberrations in the DMN have been reported in previous studies: i.e., an overall heightened activation of DMN structures during cognitive tasks was observed (Pomarol-Clotet et al., 2008; Whitfield-Gabrieli et al., 2009). DMN abnormalities have been demonstrated in siblings and other first-degree relatives of patients with schizophrenia in previous studies as well; reduced task-related suppression (Garrity et al., 2007), abnormally high functional connectivity (Whitfield-Gabrieli et al., 2009) or reduced functional connectivity (Jang et al., 2011). Possibly, less deactivation in the MFG and the PCC during reward processing may signal a failure to fully disengage from the default-mode and thus engage neural resources underlying reward-related behavior. Furthermore, these results may suggest that reward processing in siblings may be more internally-focused, since the DMN is more active during internally-focused tasks (Buckner et al., 2008).

The failure to disengage from the DMN in siblings is related to the amount of subclinical negative symptoms; we found a positive correlation between negative symptom scores and BOLD signal change in the MFG. Siblings with higher subclinical negative symptoms demonstrate even lower task-related supression during a reward processing task. This association could mean that default mode is associated with the self-referential aspect of negative symptoms. This could suggest an impaired ability to suppress attention to internal states, which might lead to a disturbance during cognitive processes. More specifically, regions of the DMN may be involved in attentional deficits in schizophrenia and symptoms such as distractibility and impaired focus on irrelevant internal and external stimuli and could interfere with motivational behavior, thinking and functioning. Heightened activation of the default mode brain regions has the potential to interfere with the effectiveness of goal-directed task performance (Sonuga-Barke and Castellanos, 2007). Since we found this association between negative symptoms and the medial frontal part of the DMN in a sample of siblings of patients with schizophrenia, who do not show behavioral symptoms on a clinical level and no behavioral impairments on reward processing, we suggest that hyperactivation of the default mode could be a endophenotype in the formation of symptoms in schizophrenia.

In addition to deactivation in DMN regions, we found deactivation in siblings compared to controls in the right insula. This area is part of the cognitive task control or salience network (Dosenbach et al., 2007; Seeley et al., 2007). This network is formed by the insula and ACC and is thought to play a pivotal role in the disengagement of the DMN and engagement of task-related networks (Menon and Uddin, 2010). Moreover, it has been hypothesized that this network may not function normally in patients with schizophrenia (White et al., 2010; Palaniyappan and Liddle, 2012). Reward processing abnormalities in schizophrenia may be a consequence of abnormal interactions between the salience network—insula and ACC—and the brain's reward processing network. Furthermore, Gradin et al. (2013) found reduced functional connectivity in patients with schizophrenia between dopaminergic midbrain regions and the right insula, which was associated with more severe positive, psychotic symptoms (Gradin et al., 2013). In a study including individuals who experience occasional mild psychotic symptoms, but are otherwise healthy, the authors found a positive correlation between connectivity in the cingulo-opercular (CO) network and positive symptoms. The cingulo-opercular network is composed of anterior insula, dorsal anterior cingulate cortex, and thalamus (Orr et al., 2014). We did not find such a correlation in healthy siblings. Our results contrast the results found in previous research; the insula was found to show decreased activity compared to healthy controls instead of less deactivation in siblings (de Leeuw et al., 2015), patients (Morris et al., 2011), and individuals with a high clinical risk (Juckel et al., 2012). These differences can possibly be explained by different methodology and the fact that our sample of siblings was particularly healthy and was especially low on subclinical positive symptoms.

As hypothesized, we did not find group differences in the predefined regions of interest in the reward consumption phase; the mPFC and the dlPFC. However, in the whole-brain analyses, we found four brain regions where siblings showed more activation than controls. Specifically, less deactivation was observed in the bilateral MFG and the PCC. Again, these regions are part of the DMN and, similar to deactivation in the anticipation phase, the findings may reflect less task-related suppression of brain activation in these areas. These results indicate that, for siblings, reduced task-related suppression of these brain areas during reward processing is present at the time of reward consumption as well as reward anticipation. In addition, siblings showed more activation in the precuneus compared to controls, this is in contrast to the findings of a previous study in patients, where patients showed increased deactivation (Garrity et al., 2007).

Taken together, our findings do not point to deficits in classical reward-related brain areas in siblings of patients with schizophrenia. Nonetheless, the results do indicate that abnormal neural responses are present during reward processing in siblings, which may suggest reward processing deficits could be partly caused by the genetic load in schizophrenia. The lack of disengagement of the DMN may reflect decreased task-related suppression during reward-related tasks for siblings, even though this is not manifest at the behavioral level. Also, since we found the same result of DMN hyperactivation during reward anticipation and reward consumption this might indicate that siblings of patients have an overall heightened DMN activity, resulting in a higher baseline level of DMN activation. Overall heightened activation of default mode structures has been reported before in patients with schizophrenia (Pomarol-Clotet et al., 2008; Whitfield-Gabrieli et al., 2009). Abnormal functioning of the DMN in schizophrenia has intriguing theoretical possibilities. As this network normally shows decreases in activation during task performance, the failure to deactivate the default mode could be relevant to disturbed cognitive processes (Sonuga-Barke and Castellanos, 2007) and might play a role in the development of symptoms in schizophrenia. Our results also point to the probability of a spectrum of abnormal network (reward regions, DMN, salience network) functioning and aberrant connectivity underlying the psychosis continuum. The results further support the relevance of the study of first-degree relatives of patients to enhance our understanding of genetic and pathogenic processes.

Future studies should shed more light on the relation between the different networks that seem disturbed in schizophrenia and relatives, such as the reward network, the DMN and the salience network. Specifically, communication between reward-related areas and other networks might be the underlying endophenotype of reward-related symptoms in schizophrenia. In addition, investigating the DMN in first-degree relatives of patients with schizophrenia may further highlight the importance of the DMN in the pathophysiology of schizophrenia and DMN abnormalities as a possible endophenotype in schizophrenia.

## Funding

LK supported by a NWO VICI grant 453-11-005. SS supported by a Medical Research Council New Investigator Award Project grant 93641 and an ERC Consolidator Award. AA was supported by a NWO VICI grant 453-11-004.

### Conflict of interest statement

The authors declare that the research was conducted in the absence of any commercial or financial relationships that could be construed as a potential conflict of interest.
