# Supplementary material for: Neural correlates of reward processing in healthy siblings of patients with schizophrenia
Source: Front Hum Neurosci. 2015 Sep 23;9:504. doi: 10.3389/fnhum.2015.00504 (PMC4585217; doi:10.3389/fnhum.2015.00504)
Supplement: Supplementary file 1 [file Table1.DOCX]

***Supplementary Material***

**Neural correlates of reward processing in healthy siblings of patients with schizophrenia**

**Esther Hanssen, MSc^1, 3^*, Jorien van der Velde, PhD^2^, Paula Gromann, MSc^1, 3^, Sukhi Shergill, MD, PhD^3^, Lieuwe de Haan, MD, PhD^4^,** **Richard Bruggeman, MD, PhD^5^, Lydia Krabbendam, PhD^1^, André Aleman, PhD^2^, Nienke van Atteveldt, PhD^1^**

^1^ Department of Educational Neuroscience and LEARN! Institute, VU University Amsterdam, Amsterdam, The Netherlands

^2^ Neuroimaging Center, University of Groningen, University Medical Center Groningen, Groningen, The Netherlands

^3^ CSI Lab, Institute of Psychiatry, Department of Psychosis Studies, King’s College London, London, United Kingdom

^4^ Department of Early Psychosis, Academic Psychiatric Centre, AMC, Amsterdam, The Netherlands

^5^ University of Groningen, University Medical Center Groningen, University Center for Psychiatry, Rob Giel Research *center*, Groningen, The Netherlands

**Supplementary Table**

Table 1

*A priori defined regions of interest (ROIs)*

|  | Talairach coordinates | | |
| --- | --- | --- | --- |
|  | x | y | z |
|  |  |  |  |
| Left VS | -10 | 5 | 4 |
| Right VS | 11 | 5 | 1 |
| VTA / SN | -1 | -20 | -10 |
| ACC | -4 | 26 | 29 |
| mPFC | 3 | 39 | 25 |
| dlPFC | 40 | 56 | 12 |

*Note* Talairach coordinates are based on Nielsen et al. (2012b)
