# Supplementary material for: Neural correlates of reward processing in healthy siblings of patients with schizophrenia
Source: Front Hum Neurosci. 2015 Sep 23;9:504. doi: 10.3389/fnhum.2015.00504 (PMC4585217; doi:10.3389/fnhum.2015.00504)
Supplement: Supplementary file 2 [file Table2.DOCX]

***Supplementary Material***

**Neural correlates of reward processing in healthy siblings of patients with schizophrenia**

**Esther Hanssen, MSc^1, 3^*, Jorien van der Velde, PhD^2^, Paula Gromann, MSc^1, 3^, Sukhi Shergill, MD, PhD^3^, Lieuwe de Haan, MD, PhD^4^,** **Richard Bruggeman, MD, PhD^5^, Lydia Krabbendam, PhD^1^, André Aleman, PhD^2^, Nienke van Atteveldt, PhD^1^**

^1^ Department of Educational Neuroscience and LEARN! Institute, VU University Amsterdam, Amsterdam, The Netherlands

^2^ Neuroimaging Center, University of Groningen, University Medical Center Groningen, Groningen, The Netherlands

^3^ CSI Lab, Institute of Psychiatry, Department of Psychosis Studies, King’s College London, London, United Kingdom

^4^ Department of Early Psychosis, Academic Psychiatric Centre, AMC, Amsterdam, The Netherlands

^5^ University of Groningen, University Medical Center Groningen, University Center for Psychiatry, Rob Giel Research *center*, Groningen, The Netherlands

**Supplementary Table**

Table 2

*Brain regions showing a main effect of the reward task in the anticipation phase*

| Cerebral Regions*  Task effect  *Large+Small Anticipation > Control Anticipation* | Hemisphere | Brodmann area | Talairach coordinates | | | Cluster size  Nr. Of voxels (mm3) |
| --- | --- | --- | --- | --- | --- | --- |
|  |  |  | x | y | z |  |
|  |  |  |  |  |  |  |
| Precentral gyrus | Right | 6 | 41 | -10 | 49 | 1635 |
| Inferior Frontal Gyrus | Right | 13 | 32 | 23 | 8 | 841 |
| Caudate | Left | - | -1 | 6 | 5 | 14292 |
| Medial Frontal Gyrus | Right | 6 | 1 | -3 | 51 | 14117 |
| Precentral Gyrus | Left | 4 | -36 | -22 | 51 | 8287 |
| Red Nucleus | Left | - | -3 | -22 | -4 | 1212 |

** Bonferroni corrected at p = .001*
