# Supplementary material for: Neural correlates of reward processing in healthy siblings of patients with schizophrenia
Source: Front Hum Neurosci. 2015 Sep 23;9:504. doi: 10.3389/fnhum.2015.00504 (PMC4585217; doi:10.3389/fnhum.2015.00504)
Supplement: Supplementary file 3 [file Table3.DOCX]

***Supplementary Material***

**Neural correlates of reward processing in healthy siblings of patients with schizophrenia**

**Esther Hanssen, MSc^1, 3^*, Jorien van der Velde, PhD^2^, Paula Gromann, MSc^1, 3^, Sukhi Shergill, MD, PhD^3^, Lieuwe de Haan, MD, PhD^4^,** **Richard Bruggeman, MD, PhD^5^, Lydia Krabbendam, PhD^1^, André Aleman, PhD^2^, Nienke van Atteveldt, PhD^1^**

^1^ Department of Educational Neuroscience and LEARN! Institute, VU University Amsterdam, Amsterdam, The Netherlands

^2^ Neuroimaging Center, University of Groningen, University Medical Center Groningen, Groningen, The Netherlands

^3^ CSI Lab, Institute of Psychiatry, Department of Psychosis Studies, King’s College London, London, United Kingdom

^4^ Department of Early Psychosis, Academic Psychiatric Centre, AMC, Amsterdam, The Netherlands

^5^ University of Groningen, University Medical Center Groningen, University Center for Psychiatry, Rob Giel Research *center*, Groningen, The Netherlands

**Supplementary Table**

Table 3

*Brain regions showing a main effect of task in the reward consumption phase*

| Cerebral Regions*  Task effect  *Outcome Win > Control Outcome* | Hemisphere | Brodmann area | Talairach coordinates | | | Cluster size  Nr. Of voxels  (mm3) |
| --- | --- | --- | --- | --- | --- | --- |
|  |  |  | x | y | z |  |
|  |  |  |  |  |  |  |
| Lingual Gyrus | Right | 18 | 3 | -67 | 2 | 145815 |
| Middle Frontal Gyrus | Right | 46 | 44 | 28 | 20 | 16882 |
| Parahippocampal Gyrus | Right | 35 | 18 | -20 | -10 | 411 |
| Superior Frontal Gyrus | Right | 8 | 13 | 35 | 48 | 641 |
| Cingulate Gyrus | Left | 31 | 0 | -34 | 31 | 4849 |
| Precuneus | Right | 7 | 2 | -68 | 34 | 2158 |
| Medial Frontal Gyrus | Left | 9 | 0 | 42 | 29 | 12690 |
| Middle Frontal Gyrus | Left | 9 | -46 | 8 | 34 | 6184 |
| Inferior Frontal Gyrus | Left | 46 | -48 | 43 | 10 | 426 |
| Inferior Frontal Gyrus | Left | 47 | -49 | 20 | -5 | 497 |
| Middle Temporal Gyrus | Left | 21 | -63 | -39 | -8 | 739 |

** Bonferroni corrected at p = .001*
