# Supplementary material for: Neural correlates of reward processing in healthy siblings of patients with schizophrenia
Source: Front Hum Neurosci. 2015 Sep 23;9:504. doi: 10.3389/fnhum.2015.00504 (PMC4585217; doi:10.3389/fnhum.2015.00504)
Supplement: Supplementary file 4 [file DataSheet1.DOCX]

***Supplementary Material***

**Neural correlates of reward processing in healthy siblings of patients with schizophrenia**

**Esther Hanssen, MSc^1, 3^*, Jorien van der Velde, PhD^2^, Paula Gromann, MSc^1, 3^, Sukhi Shergill, MD, PhD^3^, Lieuwe de Haan, MD, PhD^4^,** **Richard Bruggeman, MD, PhD^5^, Lydia Krabbendam, PhD^1^, André Aleman, PhD^2^, Nienke van Atteveldt, PhD^1^**

^1^ Department of Educational Neuroscience and LEARN! Institute, VU University Amsterdam, Amsterdam, The Netherlands

^2^ Neuroimaging Center, University of Groningen, University Medical Center Groningen, Groningen, The Netherlands

^3^ CSI Lab, Institute of Psychiatry, Department of Psychosis Studies, King’s College London, London, United Kingdom

^4^ Department of Early Psychosis, Academic Psychiatric Centre, AMC, Amsterdam, The Netherlands

^5^ University of Groningen, University Medical Center Groningen, University Center for Psychiatry, Rob Giel Research *center*, Groningen, The Netherlands

**Methods & Results: Additional statistical analyses**

A linear regression model with scan site, age and group as predictors showed no significant effect of age on any of the behavioral measures, i.e. RT in control trials (*F* (1,145) = .004, *p* = .949), RT in small reward trials (*F* (1,147) = .007, *p* = .993), RT in large reward trials (*F* (1,147) = .738, *p* = .392), accuracy in control trials (*F* (1,145) = .084, *p* = .772), accuracy in small reward trials (*F* (1,147) = .011, *p* = .915), accuracy in large reward trials (*F* (1,147) = .192, *p* = .662).

Paired sampled t-tests on the beta values during small and large reward anticipation combined showed no differences between reward sizes in the brain areas that show group differences in the insula *t* (150) = .515, *p* = .607, the posterior cingulate cortex *t* (150) = -1.123, *p* = .263, the medial frontal gyrus *t* (150) = -.274, *p* = .784 and the paracentral gyrus *t* (150) = -1.373, *p* = .172.
